# The Phosphate-Bridged Pentapalladium(II)-Containing 18-Tungsto-4-Phosphate [Pd_5_O_2_(HPO_4_)_2_(PW_9_O_34_)_2_]^16–^: Synthesis and Physicochemical Properties

**DOI:** 10.1021/acs.inorgchem.6c01326

**Published:** 2026-06-25

**Authors:** Lakita Khidtta, Ananthu Rajan, Mahmoud Elcheikh Mahmoud, Vinaya Siby, Bassem S. Bassil, Dorothea Schmidt, Nikolai Kuhnert, Pierre Bauduin, Ulrich Kortz

**Affiliations:** † School of Science, 84498 Constructor University, Campus Ring 1, Bremen 28759, Germany; ‡ ICSM, Université de Montpellier, CEA, CNRS, ENSCM, Marcoule, Bagnols sur, Cèze Cedex 30207, France

## Abstract

The pentapalladium­(II)-containing 18-tungsto-4-phosphate [Pd_5_O_2_(HPO_4_)_2_(PW_9_O_34_)_2_]^16–^ (**Pd**_**5**_) has been synthesized in a simple one-pot procedure reacting Pd^2+^ ions with the trilacunary precursor [*A*-α-PW_9_O_34_]^9–^ at room temperature in aqueous dimethylarsinate buffer with some free phosphate ions. The polyanion **Pd**_**5**_ comprises a [Pd_5_O_2_(HPO_4_)_2_]^2+^ assembly sandwiched between two [*A*-α-PW_9_O_34_]^9–^ units, and it was isolated as a hydrated sodium salt, Na_16_[Pd_5_O_2_(HPO_4_)_2_(PW_9_O_34_)_2_]·25H_2_O (**Na-Pd**_**5**_), which was characterized in the solid state by single-crystal XRD, FT-IR, TGA, and elemental analysis. In solution, **Na-Pd**_**5**_ was characterized by ^31^P NMR, ESI-MS, UV–vis, and small- and wide-angle X-ray scattering (SWAXS). Furthermore, **Na-Pd**_**5**_ was employed as a precatalyst immobilized on SBA-15-apts for the hydrogenation of *o*-xylene, which showed excellent activity and recyclability.

## Graphical abstract

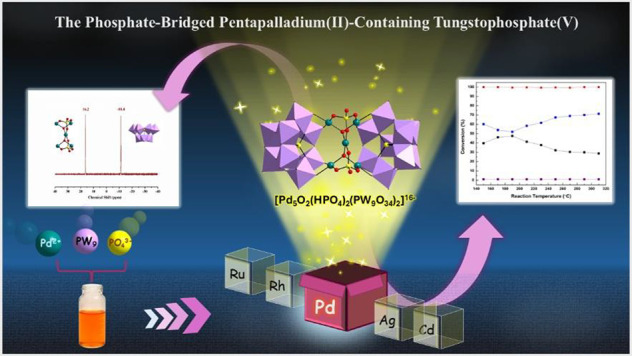

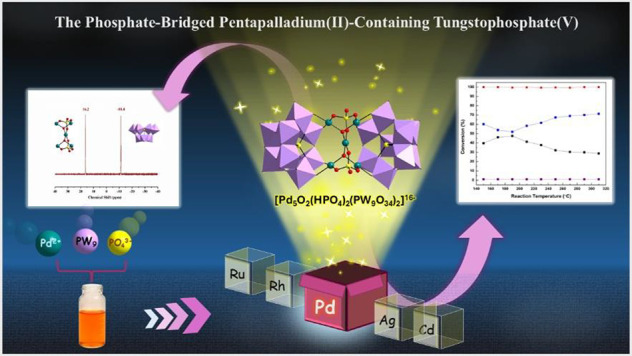

## Introduction

The interplay of metal ions and oxo ligands in precise arrangements, typically involving edge- or corner-shared MO_6_ octahedra with M being an early *d*-block metal (addenda) ion in a high oxidation state (e.g., W^6+^, Mo^6+^, V^5+^) leads to the formation of polyoxometalates (POMs).^1^ POMs are an intriguing family of discrete metal–oxo anions with enormous structural and compositional variety and manifold physicochemical properties.^2^ POMs can be subdivided in isopoly and heteropolyanions, the latter with the general formula [X_m_M_n_O_l_]^q–^ (M = addenda metal ion, X = heteroatom, a common example is [PW_12_O_40_]^3–^), whereas isopolyanions do not have any heteroatom (e.g., [V_10_O_28_]^6–^). Some heteropolyanions can form lacunary (vacant) derivatives by removal of one or more MO_6_ octahedra, e.g., [PW_11_O_39_]^7–^.^1^ Lacunary POMs have a higher charge density than their plenary analogues (especially at the vacant site) and can be seen as polydentate ligands, and hence are reactive and nucleophilic with oxophilic cations of the *d* or *f* or even *p*-block (or cationic organometallic entities).^3^ The trilacunary Keggin-based 9-tungstophosphate precursor [*A*-α-PW_9_O_34_]^9–^ has been extensively studied with metal ions as well as with organometallic groups, resulting in products with interesting properties, e.g., in the field of catalysis.^4^

Palladium-containing (or noble metal, in general) POMs are less common than 3*d* metal- and lanthanides-containing derivatives, due to the lower reactivity of noble metal ions (largely independent of oxidation state) with lacunary heteropolyanions. The first structurally characterized palladium­(II)-containing POM was the isopoly-10-tungstate [Pd^II^_2_W_10_O_36_]^8–^, reported by Angus-Dunne and coworkers in 1994.^5^ The reactivity of Pd^2+^ toward heteropolytungstate lacunary derivatives has been studied further since then. The reactivity of Pd^2+^ ions with lone-pair-containing trilacunary Keggin ions has been investigated for several [XW_9_O_33_]^n–^ systems (X = Sb^III^, As^III^, Te^IV^, Bi^III^). The first structurally characterized example was the Sb^III^ derivative [Cs_2_Na­(H_2_O)_10_Pd^II^_3_(α-Sb^III^W_9_O_33_)_2_]^9–^ reported in 2004,^6a^ followed in 2005 by the As^III^ analogues [Na_2_(H_2_O)_2_Pd^II^WO­(H_2_O)­(α-As^III^W_9_O_33_)_2_]^10–^ and [Cs_2_Na­(H_2_O)_8_Pd^II^_3_(α-As^III^W_9_O_33_)_2_]^9–^.^6b^ In 2009, an example with Bi^III^ as heteroatom was reported, [Pd^II^_3_(H_2_O)_9_Bi_2_W_22_O_76_]^8–^, in which three palladium­(II) ions are grafted on the polyanion surface.^6c^ The Te^IV^-containing analogue [Pd^II^_3_(Te^IV^W_9_O_33_)_2_]^10–^ was reported in 2012.^6d^ As concerns the interaction of Pd^2+^ ions with classical trilacunary Keggin ions with no lone pair on the heteroatom, such as the trilacunary 9-tungstosilicate [*A*-α-SiW_9_O_34_]^10–^, the dipalladium­(II)-containing sandwich-type ion [Cs_2_K­(H_2_O)_7_Pd^II^_2_WO­(H_2_O)­(*A*-α-SiW_9_O_34_)_2_]^9–^ was reported in 2004.^7^ With respect to the trilacunary 9-tungstophosphate [*A*-α-PW_9_O_34_]^9–^, in 1986 Knoth and coworkers synthesized the tripalladium­(II)-containing sandwich-type ion [Pd^II^_3_(*A*-α-PW_9_O_34_)_2_]^12–^, characterized by elemental analysis, but no crystal structure analysis.^8^ In 2000, Kuznetsova and coworkers studied the compound with IR and NMR spectroscopy and investigated its activity for liquid phase hydrocarbon oxidation.^9^ In 2009 Villanneau et al. reported the X-ray structure of the dipalladium­(II)-containing [Pd^II^_2_(WO­(H_2_O))­(*A*,α-PW_9_O_34_)_2_]^10–^, and they also reported the monopalladium­(II) analogue [Pd^II^{WO­(H_2_O)}_2_{*A*,α-PW_9_O_34_}_2_]^8–^ and the tripalladium­(II) analogue [Pd^II^_3_{*A*,α-PW_9_O_34_}_2_]^12–^, as based on solution NMR.^10^ In 2021, Patel reported the crystal structure of the tripalladium­(II) sandwich-ion [Pd^II^_3_(PW_9_O_34_)_2_]^11–^.^11^ In addition to these examples, a variety of other Pd^II^-based polyoxotungstates derived from different lacunary precursors have also been reported.^12^

Despite the evident complexity in the synthesis and structural elucidation of noble metal-containing POMs, their promising catalytic potential continues to drive research in this area.^13^ Palladium is a highly effective catalyst for hydrogenation and oxidation reactions,^14a–14c^ first observed when Davy found that palladium and platinum enabled flameless combustion.^14d^ POMs allow for precise tuning of active sites and hence are ideal models to study single-site catalysis.^15^ Significant research has demonstrated that palladium­(II)-containing polyoxotungstates exhibit efficient catalytic activity: [Pd_4_(As_2_W_15_O_56_)_2_]^16–^ for Suzuki-Miyaura coupling,^12g^ [γ-H_2_SiW_10_O_36_Pd_2_(OAc)_2_]^4–^ for nitrile hydration in water,^13a^ and the Tourné-type sandwich-ion [WZnPd_2_(ZnW_9_O_34_)_2_]^12–^ for H_2_O_2_ oxidations,^13b^ among numerous other examples.

Hence, we decided to research further on the synthesis of novel palladium­(II)-containing heteropolytungstates with interesting catalytic activity.

## Experimental Section

### Materials and Physical Measurements

The trilacunary tungstophosphate precursor Na_9_[*A*-α-PW_9_O_34_]·7H_2_O was synthesized according to a published procedure,^16^ and its identity was confirmed by infrared spectroscopy. All other reagents were used as received, without additional purification. Fourier-transform infrared spectra (FT-IR) were recorded using KBr pellets on a SHIMADZU FT-IR spectrophotometer operating between 4000–400 cm^–1^ (Figure S2). The peak intensities are indicated using the following abbreviations: w, weak; m, medium; s, strong; sh, shoulder. The ^31^P NMR spectra were recorded in H_2_O at room temperature using a JEOL ECX 400 MHz spectrometer (D_2_O was used as locking solvent). The resonance frequency for ^31^P NMR was 162.138 MHz and the chemical shifts are reported with respect to 85% H_3_PO_4_ as reference. The thermal stability of **Na-Pd**_**5**_ (Figure S7) was determined using thermogravimetric analysis (TGA) on a TA Instruments Q600 device, with heating from room temperature to 600 °C at a rate of 5 °C/min under a nitrogen atmosphere. The elemental analyses for Pd, W, and P were performed by Zentrallabor, Technische Universität Hamburg, Am Schwarzenberg-Campus 1, 21073 Hamburg. The sodium analyses were performed in house by atomic absorption (AA) spectroscopy on a Varian SpectrAA 220 AA instrument. Ultraviolet–visible (UV–vis) measurements were carried out with a Varian Cary 100 Bio UV–vis spectrophotometer across the 200–800 nm wavelength range, using 1 cm quartz cuvettes. PXRD measurements were carried out on a Rigaku Miniflex 600 diffractometer using Cu Kα radiation (λ = 1.541838 Å) operated at 40 kV and 15 mA. Data were collected over a 2θ range of 3–80° with a step size of 0.01° and a scan rate of 2° min^–1^.

### Synthesis of Na_16_[Pd_5_O_2_(HPO_4_)_2_(PW_9_O_34_)_2_]·25H_2_O (**Na-Pd**_**5**_)

Na_9_[*A*-α-PW_9_O_34_]·7H_2_O (0.513 g, 0.200 mmol) was dispersed in 6 mL of 1 M sodium dimethylarsinate (cacodylate) buffer (pH 7). Pd­(OAc)_2_ (0.123 g, 0.55 mmol) and Na_3_PO_4_ (0.032 g, 0.200 mmol) were then added to this dispersion and the mixture was stirred for 2 days at room temperature. Over time, the mixture gradually dissolved, resulting in the formation of a clear dark-red solution. After 2 days, the pH of this solution was adjusted to 8 using 6 M NaOH, and the stirring was continued for 10 more hours. The resulting solution was then filtered and the filtrate kept for evaporation in an open vial. Dark-red needle-shaped crystals (Figure S1) were collected after 1–2 weeks and dried in air. Yield: 0.36 g (60%, based on W). IR data (KBr pellet) (in cm^–1^): 1073 (m), 1012 (m), 937 (sh), 840 (m), 772 (m), 697 (m), 611 (m), 515 (m). Elemental analysis, calcd (found) for **Na-Pd**_**5**_: Na, 6.1 (6.8); W, 54.8 (54.6); Pd, 8.8 (9.1); P, 2.1 (1.7).

### X-ray Crystallography

A **s**ingle crystal of **Na-Pd**_**5**_ was mounted in a Hampton cryoloop coated with Paratone-N oil to prevent water loss. For data collection at 100 K a Rigaku XtaLAB Synergy Dualflex HyPix single-crystal diffractometer was used equipped with a sealed molybdenum tube and graphite monochromator with Mo Kα radiation (λ = 0.71073 Å) operated via the CrysAlisPro software package (CrysAlis^Pro^ Software System, Version 1.171.38.41, Rigaku Oxford Diffraction 2022). A multiscan absorption correction was performed using the ABSPACK program.^17^ The structural solutions were found using direct techniques, followed by refinement with the full-matrix least-squares approach [Σw (|F_o_|^2^ – |F_c_|^2^)^2^], using anisotropic thermal parameters for all atoms included in the model except for oxygen and disordered counter cations. The SHELXL-2014 software package (Bruker) was used for structure solution and refinement.^18^ The Diamond software, version 3.2 (copyright, Crystal Impact GbR) was used to generate crystal structure images. The details of the crystal data and structure refinement for **Na-Pd**_**5**_ are shown in Table 1. The Cambridge Crystallographic Data file CSD-2471680 contains the supplementary crystallographic data for this paper. These data can be obtained free of charge from The Cambridge Crystallographic Data Centre via www.ccdc.cam.ac.uk/.

**Table 1:** Single Crystal Data and Structure Refinement Parameters for **Na-Pd_5_**

Compound	**Na-Pd**_**5**_
Empirical formula^a^	H_52_O_103_Na_16_P_4_Pd_5_W_18_
Formula weight^a^, g mol^–1^	6033.43
Crystal system	Triclinic
Space group	P1̅
*a* (Å)	13.38400(10)
*b* (Å)	19.19770(10)
*c* (Å)	24.5094(2)
α (deg)	74.1000(10)
β (deg)	74.4860(10)
γ (deg)	84.2420(10)
Volume, Å^3^	5833.41(8)
Z	2
*D*_calc_, g/cm^3^	3.435
Crystal size, mm × mm × mm	0.26 × 0.09 × 0.04
Absorption coefficient, mm^–1^	18.633
F(000)	5348
2θ range for data collection, deg	2.3640 to 33.9660
Index ranges	–20 ≤ *h* ≤ 20, −30 ≤ *k* ≤ 30, −38 ≤ *l* ≤ 35
Reflections collected	446193
Independent reflections	43818
R(int)	0.120
Data/restraints/parameters	43818/0/842
Goodness-of-fit on F^2^	1.007
R_1_,^b^ *w*R_2_ ^c^ (*I* > 2σ(*I*))	R_1_ = 0.0797, *w*R_2_ = 0.1666
R_1_,^b^ *w*R_2_ ^c^ (all data)	R_1_ = 0.1069, *w*R_2_ = 0.1771
Largest diff peak and hole, e Å^–3^	5.84 and −6.02

a True formula unit and molar mass of **Na-Pd**_**5**_ as obtained from elemental analysis on bulk material.

b R_1_ = ∑||F_o_| – |F_c_||/∑|F_o_|.

c wR_2_ = [∑*w* (F_o_^2^ – F_c_^2^)^2^/∑*w* (F_o_^2^)^2^]^1/2^.

### ESI Mass Spectrometry

High-resolution mass spectra were recorded using a Bruker Daltonics QTOF Impact HD mass spectrometer employing both negative and positive electrospray ionization modes. The spectrometer was fitted with an ESI source and external calibration was achieved with 10 mL of 0.1 M sodium formate solution. The instrument ion source and tubing were rinsed with methanol. Calibration was carried out using the enhanced quadratic calibration mode. All MS measurements were performed in both negative and positive ion modes. Samples were measured as direct infusions at a concentration of 10 μg/mL in deionized water at a flow rate of 180 μL/min. Samples were prepared by dissolving 1 mg of **Na-Pd**_**5**_ in 1 mL of deionized water followed by a 1:100 dilution. Spectral simulation was carried out in Data Analysis 4.1 (Bruker Daltonics, Bremen).

### Small- and Wide-Angle X-ray Scattering (SWAXS)

SWAXS measurements using Mo radiation (λ = 0.071 nm) were performed on a custom-built bench by XENOCS. Solutions of **Na-Pd**_**5**_, with and without detergents in water, were loaded into 2 mm quartz capillaries. The scattered beam was recorded using a large online scanner detector (diameter: 345 mm, MAR Research). Collimation was achieved using a 12:∞ multilayer Xenocs mirror (optimized for Mo radiation) coupled with two sets of scatterless FORVIS slits, providing a 0.8 × 0.8 mm X-ray beam at the sample position. The scattered intensities are presented as a function of the scattering vector magnitude, q = [(4π)/λ] sin­(θ/2), where λ is the incident radiation wavelength and θ is the scattering angle. Standard corrections for background subtraction (including empty capillary and detector noise) and intensity normalization were applied, using a high-density polyethylene film as a reference. The experimental resolution was Δq/q = 0.05. Silver behenate in a sealed capillary was used as the calibration standard for the scattering vector. All spectra were corrected by subtracting signals from the empty capillary, and the final scattering intensities are given in absolute units (cm^–1^). All experiments were conducted at 22 ± 2 °C.

### Cloud Point Determination

Cloud point measurements were performed in a 1 mL cuvette under magnetic stirring. The cuvette was immersed in a water bath with controlled heating. The cloud point of octyl tetraethylene glycol ether (C8E4) at a concentration of 60 mM in water was determined to be 42 °C in agreement with the literature. **Na-Pd**_**5**_ solutions were prepared in 60 mM C8E4 to maintain a constant detergent concentration. Cloud point variations were initially detected by visual observation, as some **Na-Pd**_**5**_-containing solutions transitioned from cloudy to transparent. The experiment was then continued to measure the new cloud point temperature. The thermometer used had an accuracy of ±0.5 °C. Octyl tetraethylene glycol ether (CAS: 19327-39-0) was purchased from Sigma-Aldrich. The cloud point evolution was measured for concentrations of **Na-Pd**_**5**_ up to 10 mM.

## Results and Discussion

### Synthesis and Structure

We have synthesized the novel pentapalladium­(II)-containing 18-tungsto-4-phosphate [Pd_5_O_2_(HPO_4_)_2_(PW_9_O_34_)_2_]^16–^ (**Pd**_**5**_) by the interaction of Pd^2+^ ions with the trilacunary POM precursor [*A*-α-PW_9_O_34_]^9–^ (**PW_9_**) at room temperature in a 1 M sodium cacodylate solution (pH 8) in the presence of phosphate. The title compound crystallized as a hydrated sodium salt, Na_16_[Pd_5_O_2_(HPO_4_)_2_(PW_9_O_34_)_2_]·25H_2_O (**Na-Pd**_**5**_). Single-crystal XRD measurements of **Na-Pd**_**5**_ revealed that the polyanion crystallizes in the triclinic space group *P*1̅ (Table 1). The structure of **Pd**_**5**_ comprises a central penta-palladium core capped by two hydrogen phosphate ions [Pd_5_O_2_(HPO_4_)_2_]^2+^ which is sandwiched between two trilacunary [*A*-α-PW_9_O_34_]^9–^ units, resulting in an assembly with a 16– charge (Figure 1). All Pd^2+^ ions are in square-planar coordination geometry in two structurally inequivalent positions, four basal sites and an apical one (Figure 1, right). The apical Pd^2+^ ion is coordinated to an oxygen from each of the two bridging HPO_4_ groups (Pd_a_–O = 2.030(11) Å) and to two μ_3_-oxo ligands each bridging to two basal Pd^2+^ centers (Pd_a_–O = 1.988(11)–2.007(10) Å). On the other hand, the four basal Pd^2+^ ions are coordinated each to two oxo ligands of two WO_6_ units (Pd_b_–O = 1.996(10)–2.042(12) Å), an oxygen atom of the bridging HPO_4_ group (Pd_b_–O = 2.002(13)–2.024(11) Å), and the two aforementioned μ_3_-oxygens (Pd_b_–O = 1.961(10)–1.980(10) Å). BVS for μ_3_-oxygen range from 1.801–1.802 (Table S1) hence not protonated. The two phosphate caps have a terminal hydroxo group each, with BVS range of 1.238–1.315 (Table S3). The BVS values for different types of μ_3_ and μ_2_ bridging oxygens in the central core are presented in Tables S1 and S2. BVS values for all five palladium ranges from 2.198–2.276 suggesting that all Pd ions are in +2 oxidation state.^19^ Overall, [Pd_5_O_2_(HPO_4_)_2_(PW_9_O_34_)_2_]^16–^ has idealized C_2v_ symmetry, with the C_2_ axis passing through the apical Pd^2+^ ion and being perpendicular to the idealized plane formed by the four basal Pd^2+^ ions (Figure 1).

**Figure 1:**
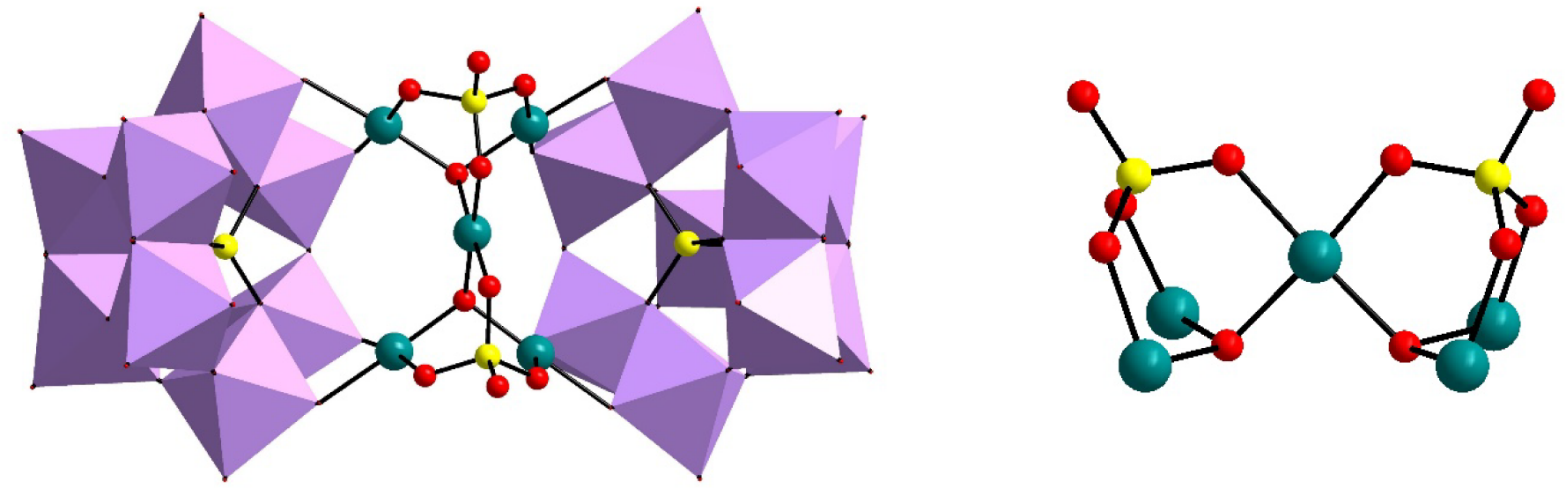
Combined polyhedral/ball-and-stick representation of **Pd_5_** (left) and the central **Pd_5_** core showing the four basal and one apical Pd^2+^ ions (right). Color code: WO_6_, lavender octahedra; P, yellow; Pd, teal, and O, red balls.

The optimized synthetic procedure has a stoichiometric ratio Pd^2+^:**PW_9_**:PO_4_^3–^ of 5:2:2, which resulted in the highest yield of pure, crystalline material. Although the presence of phosphate in the reaction mixture is essential for obtaining a pure product, the compound was originally obtained as a byproduct in the absence of phosphate, most probably due to decomposition of **PW_9_** and release of PO_4_^3–^ ions *in situ*. The role of the cacodylate as reaction medium is also important, as other buffers at the same pH did not result in the title compound and the elemental analysis confirms that its content in the isolated material is below 0.2%, consistent with the X-ray diffraction results and indicates no structural incorporation. Moreover, the pH adjustment step is crucial, as the reaction proceeds only in a cacodylate buffer at pH 7 followed by adjustment to pH 8 with NaOH; any attempt to perform the reaction directly in a pH 8 cacodylate buffer did not yield the product. Finally, room temperature is needed for the successful isolation of **Na-Pd**_**5**_ as a crystalline salt; any heating of the reaction mixture led to an amorphous solid that could not be identified.

### ^31^P NMR Spectroscopy

The solution behavior and stability of **Pd**_**5**_ was examined by ^31^P NMR spectroscopy in H_2_O at room temperature. The spectrum exhibits the expected two singlets at 16.2 and −11.4 ppm, respectively (Figure 2). The peak at −11.4 ppm can be assigned to the P atom in the **PW_9_** unit of the structure whereas the peak at 16.2 corresponds to phosphate groups capping the central Pd_5_-oxo core. The spectrum remained unchanged for around 30 min, after which the peak for free phosphate starts to appear (Figure S3). Similar shifts for phosphate groups capping polyanions have been reported in the literature, for example the phosphate-capped 12-palladate [ZnPd^II^_12_P_8_O_35_(OH)_5_]^9–^ exhibits a singlet at 15.8 ppm in ^31^P NMR,^20^ and the sandwich-type [Pd^II^_3_(PW_9_O_34_)_2_]^12–^ ion exhibits a singlet at −12.2 ppm.^10^ The solution stability of **Na-Pd**_**5**_ was assessed by time dependent ^31^P NMR spectroscopy in water (Figure S3) and sodium cacodylate solutions (Figures S4 and S5). In water, **Na-Pd**_**5**_ retains its characteristic two resonances for up to ca. 2 h, whereas pH- and time-dependent studies in sodium cacodylate buffer show improved stability, particularly under neutral to mildly basic conditions.

**Figure 2:**
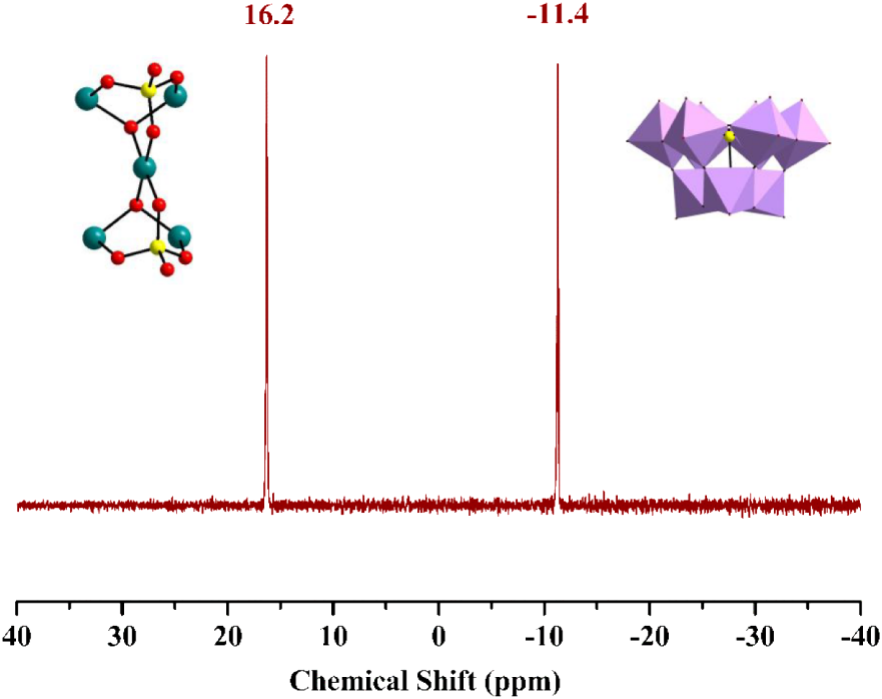
^31^P NMR spectrum of **Na-Pd_5_** recorded at room temperature in H_2_O. The structural fragments containing the relevant P atom are indicated next to the peaks.

### UV–Vis Spectroscopy

The stability of **Na-Pd**_**5**_ was investigated using UV–vis spectroscopy (Figure S6). The UV–vis spectrum of **Na-Pd**_**5**_ (5 × 10^–5^ M) recorded in H_2_O exhibits an intense absorption band at 278 nm in the ultraviolet region, attributed to ligand-to-metal charge transfer (LMCT) from oxygen π orbitals to the vacant d orbitals of the W^6+^ centers. A weaker band observed around 474 nm in the visible region is assigned to d–d transitions of the Pd^2+^ centers, likely associated with Pd–O coordination in a square-planar geometry. Time-dependent UV–vis measurements showed a progressive shift of the LMCT band to lower wavelengths (278 to 275 nm) starting after 1 h, indicating structural changes in solution. Consistently, time-dependent NMR spectra (Figure S4) confirmed decomposition after 1 h, demonstrating stability in water, followed by gradual degradation.

### ESI Mass Spectrometry

The title polyanion salt **Na-Pd**_**5**_ was analyzed by high-resolution ESI mass spectrometry in negative-ion mode in aqueous solution. The resulting full-scan mass spectrum, shown in Figure 3, exhibits a broad envelope of isotope peaks centered at *m*/*z* 1066. With a distance of 0.2 Da between isotope peaks a charge state of 5– can be assigned. A second smaller peak envelope is observed around *m*/*z* 1339 with a distance of 0.25 Da between isotope peaks suggesting an overall charge of the gas phase ion of 4–.

**Figure 3:**
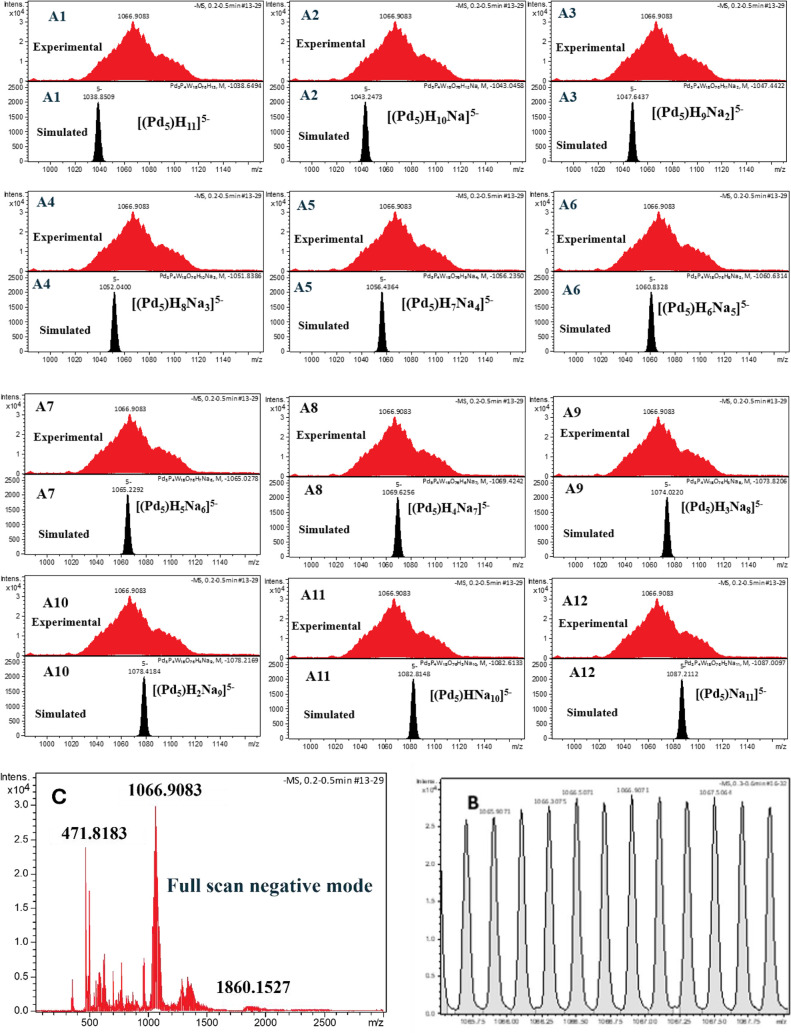
ESI mass spectra in negative ion mode of **Na-Pd_5_**; **A1–A11** upper panes experimental spectrum centered around *m*/*z* 1066; lower panes simulated spectra with varying cation composition H*_x_* Na*_y_* (x + y = 11); **B** expanded region of full scan spectrum around *m*/*z* 1066 illustrating a charge state of 5–; **C** full scan spectrum.

The experimental spectrum is a result of varying cation stoichiometry balancing the charge of the **Pd**_**5**_ anion, resulting in an overlap of multiple species in the gas phase, each contributing to a local intensity maximum of the envelope spanning 100 Da. Simulation of the spectrum assuming an elemental composition of **Pd**_**5**_ (with a 16– charge) as shown by single crystal XRD as was carried out for the system [(**Pd**_**5**_)­Na_*x*_H_*y*_]^5–^ seen by ESI-MS with systematic variation of cation stoichiometry. Assuming varying compositions of Na_*x*_H_*y*_ with x + y = 11 and x and y varying from 0 to 11 results in individual spectra in agreement with subsections of the experimental data set. Two selected simulations of [(**Pd**_**5**_)­Na_3_H_8_]^5–^ and [(**Pd**_**5**_)­Na_5_H_6_]^5–^ are shown in Figure 3. The second minor envelope centered around *m*/*z* 1339 corresponds according to comparison with simulated data to the anions with four negative charges.

### Small- and Wide-Angle X-ray Scattering (SWAXS)

The physicochemical properties of **Pd**_**5**_ in water were investigated using small-angle X-ray scattering (SAXS) to assess polyanion stability and structural integrity in this solvent. SAXS profiles of **Pd**_**5**_ in water at different concentrations (5, 10, and 30 mM), and in the presence of sodium chloride (100 mM), are shown in Figure 4. **Pd**_**5**_ exhibits excess scattering in the 0.02–0.8 Å^–1^ range, with an upturn in intensity at low q, characteristic of globular objects approximately 1 nm in size, consistent with the expected dimensions of **Pd**_**5**_. The observed scattering primarily arises from the electron density contrast between **Pd**_**5**_ containing the heavy elements Pd and W, and the surrounding water. To further characterize the scattering, simulations of the scattered intensity were performed using the known atomic coordinates of **Pd**_**5**_. PDB files were generated from the corresponding CIF crystal structure, and the SAXS simulations were carried out using the CRYSOL software,^21^ which employs a multipole expansion of the scattering amplitudes to compute the spherically averaged intensity profile. A comparison between the simulated (dashed lines) and experimental SAXS spectra is presented in Figure 4. A scaling factor and background correction (due to solvent scattering) were applied to the simulated spectra to fit the experimental spectra at 5 and 30 mM **Pd**_**5**_ in 100 mM NaCl solution.

**Figure 4:**
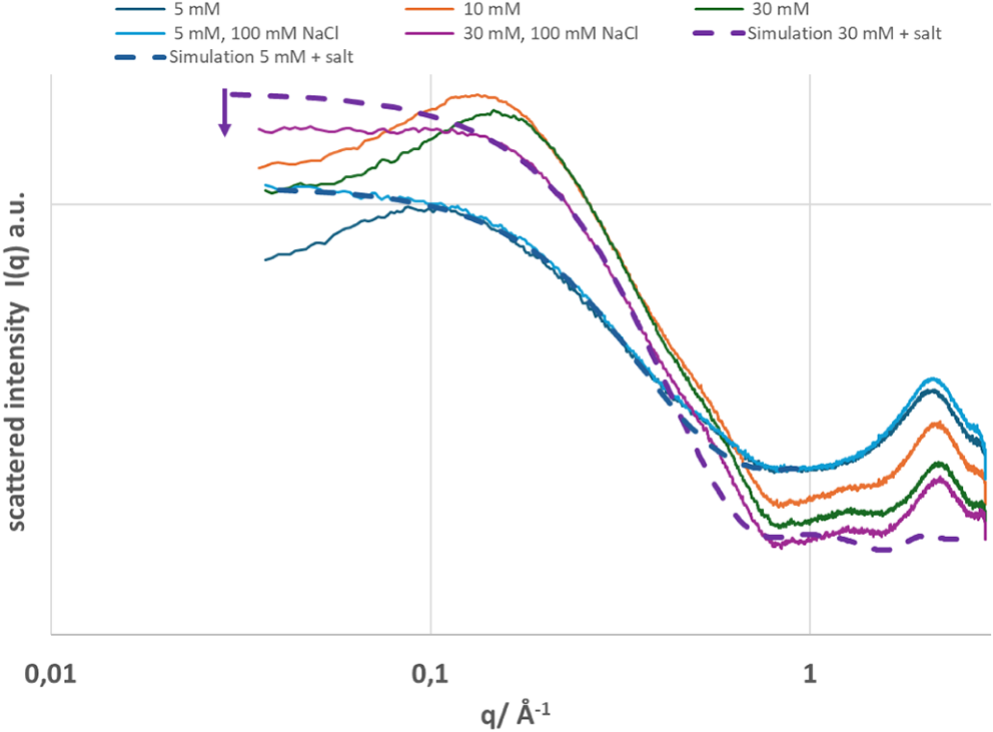
SWAXS spectra of **Na-Pd_5_** in water at different concentrations (5, 10, and 30 mM) and in the presence of sodium chloride (100 mM) to investigate electrostatic repulsions between the **Pd_5_** ions. This analysis aims to confirm the charged nature of **Pd_5_** in aqueous solution, specifically the dissociation of its counter cations. The dashed lines represent simulated curves obtained using the program Crysol, based on atomic coordinates derived from the single-crystal XRD structure. These simulations are scaled to match the experimental SWAXS data, and a background contribution is included in the fitting process.

Overall, good agreement is observed between the simulated and experimental data, particularly at a concentration of 5 mM. At 30 mM, the experimental spectra show a slight decrease in intensity compared to the simulation for *q* < 0.2 Å^–1^ (see the arrow in Figure 4), likely reflecting interpolyanion electrostatic repulsion in water, as expected for an anionic species. This decrease in low-q intensity thus confirms the charged nature of **Pd**_**5**_ in aqueous solution, likely due to countercation (Na^+^) dissociation in solution. The trend is even clearer at higher concentrations without added salt, where the reduction in low-q intensity is more pronounced at 10 and 30 mM, confirming stronger electrostatic repulsions between polyanions. Moreover, a broad interaction peak centered around 0.13 and 0.15 Å^–1^ is observed experimentally at 10 and 30 mM, respectively. This peak, which shifts to higher q (smaller distances) with increasing concentration, is consistent with enhanced intercluster correlations driven by electrostatic interactions. A slight discrepancy between the simulated and experimental spectra is noted in the 0.4 < *q* < 0.7 Å^–1^ region, where the experimental data show excess scattering, suggesting internal correlations within **Pd**_**5**_. This difference may arise from conformational variations between the crystalline and fully solvated states.

Nevertheless, the overall scattering envelope of the simulated spectra captures the main features of the experimental data, indicating that the size and shape of **Pd**_**5**_ in water are well reproduced by the model and that the polyanion retains its structural integrity in solution.

The polyanion salt **Na-Pd**_**5**_ was also investigated for its potential superchaotropic character, a property that emerges in inorganic nanometer-sized charged species when their charge density is typically below ∼12 charges/nm^3^, resulting in a tendency to bind to hydrophilic, hydrated entities (molecules or surfaces).^22^ The cloud point of C8E4 was measured upon addition of **Na-Pd**_**5**_ (up to 30 mM), following a previously reported procedure, to assess this property.^23^ No change in the cloud point was observed, as expected given the relatively high charge density of **Pd**_**5**_ (∼16 charges/nm^3^), which lies above the superchaotropic threshold.

### Hydrogenation of *o*-Xylene Using Supported Pd_5_ as Precatalyst

The POM salt **Na-Pd**_**5**_ was dissolved in water and subsequently immobilized onto mesoporous SBA-15 cationically functionalized with 3-aminopropyltriethoxysilane (SBA-15-apts), following an established procedure.^24^ The resulting supported POM was calcined in air at 450 °C for 5 h to afford a thermally robust material containing 1 wt % Pd. For all catalytic hydrogenation runs, 450 mg of the calcined material was used. The fixed-bed reactor was packed with 11 g of silicon carbide (SiC, 120 grit) at the base, followed by 450 mg of the calcined material, with an additional 3 g of SiC placed on top to stabilize the bed. The thermocouple was positioned directly within the catalyst zone to ensure accurate temperature control during the catalytic reaction. This immobilized system was evaluated for the hydrogenation of *o*-xylene under continuous flow conditions at 28 bar H_2_ in a fixed-bed reactor. As shown in Figure 5, the catalyst based on the **Pd**_**5**_ precursor exhibited quantitative hydrogenation of *o*-xylene (≥98% conversion) across a wide temperature range (150–310 °C). Remarkably, the *trans*-1,2-dimethylcyclohexane (*trans*-1,2-DMCH) isomer was the major product at all temperatures, indicating that thermodynamically favored surface equilibration dominates the reaction pathway even at lower thermal input. This is in contrast to classical behavior where *cis*-1,2-dimethylcyclohexane (*cis*-1,2-DMCH) selectivity prevails at lower temperatures due to kinetic control.^25^ Control experiments using neat SBA-15-apts and SiC under identical conditions resulted in negligible conversion, confirming the essential role of **Pd**_**5**_ as catalyst precursor. Control experiments demonstrate that neither Pd­(NO_3_)_2_-SBA-15 nor physically mixed **PW_9_** + PO_4_^3–^ + Pd^2+^ + SBA-15 reproduces the activity of the **Na-Pd**_**5**_ derived catalyst under identical conditions. These findings suggest that the preassembled **Pd**_**5**_ precursor is beneficial for generating the catalytically active phase after calcination and reaction. The catalyst is therefore best described as a Pd-POM-derived supported material rather than intact molecular Pd_5_ (Figure S8). In 2019 Kortz’s group demonstrated that discrete cuboid polyoxopalladate­(II) polyanions of the type {Pd^II^_13_L_8_} and {MPd^II^_12_L_8_} (M = 3d metal ion, L = capping group) can serve as discrete precursors for neat Pd^0^ nanoparticles or mixed-metal M^0^Pd_12_^0^ alloy-type nanoparticles highly efficient for hydrogenation catalysis and that the nature of the capping group as well as the central metal ion guest are crucial.^26^ Furthermore, in 2022 the same group further elaborated on the cuboid-shaped polyoxopalladates­(II), and were also able to modulate hydrogenation performance, depending on the composition of the starting discrete {MPd^II^_12_L_8_} polyanion.^27^

**Figure 5:**
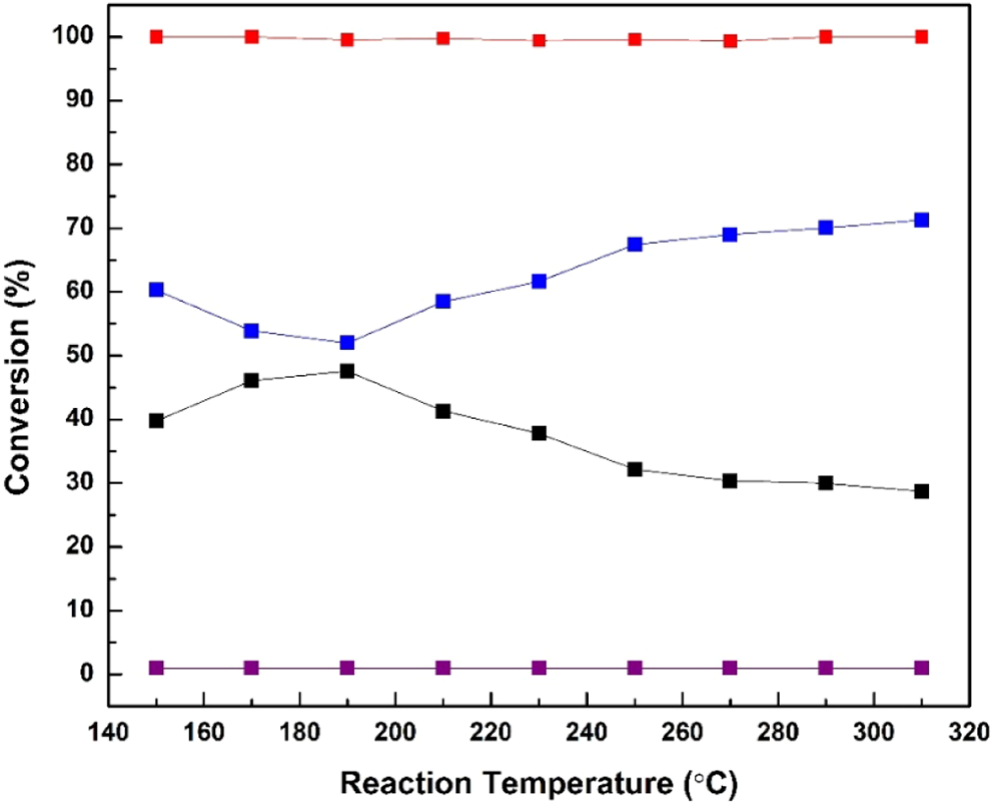
Hydroconversion of *o-*xylene to 1,2-dimethylcyclohexane (1,2-DMCH) over **Na-Pd_5_** supported on SBA-15-apts at various temperatures. Reaction conditions: 28 bar H_2_, flow rate = 0.05 mL/min. Total conversion (red), conversion to *cis*-1,2-DMCH (black) and *trans*-1,2-DMCH (blue), and the SiC control (purple). The SBA-15-apts control showed negligible activity.

To investigate the role of residence time on hydrogenation selectivity, flow rate-dependent experiments were performed at 310 °C (Figure S10). As the *o*-xylene flow rate increased from 0.05 to 0.40 mL/min, both total conversion and the *cis*/*trans*-selectivity decreased, consistent with diffusion-limited substrate activation. This behavior is characteristic of Langmuir–Hinshelwood type mechanisms, wherein both hydrogen and substrate adsorb on the catalyst surface and undergo surface mediated transformation.^28^ Shorter contact times reduce the opportunity for complete hydrogenation and surface isomerization, resulting in lowered activity and selectivity. The stability and recyclability of the catalyst based on the **Pd**_**5**_ precursor was evaluated over five consecutive reaction cycles at 150 °C and 310 °C (Figure 7). No significant decrease in conversion or isomer distribution was observed, confirming the thermal, structural, and operational robustness of the catalyst.

To gain insight into the structural evolution of the catalyst FT-IR (Figure S9) and powder X-ray diffraction (PXRD) (Figure 6) analysis was performed before and after catalysis, The formation of metallic Pd nanoparticles is more clearly supported by PXRD. Prior to catalysis, the supported material displayed only the broad amorphous reflection characteristic of SBA-15-apts, indicating the absence of detectable crystalline Pd-containing domains. After catalysis, new reflections emerged, indicating the formation of crystalline Pd-containing species during catalyst activation and reaction. In particular, the reflection at 2θ ≈ 40.3° can be assigned to the (111) plane of face-centered cubic metallic Pd (Pd^0^), while the higher-angle reflection near ∼68–70° is consistent with the Pd^0^ (220) plane. Additional reflections may arise from oxidized Pd species and/or Pd-W-containing phases. These observations indicate structural transformation of the **Na-Pd_5_** precursor under catalytic conditions and suggest that the catalytically active material is best described as a Pd/POM-derived supported catalyst rather than intact molecular **Na-Pd**_**5**_ Figure 7.

**Figure 6:**
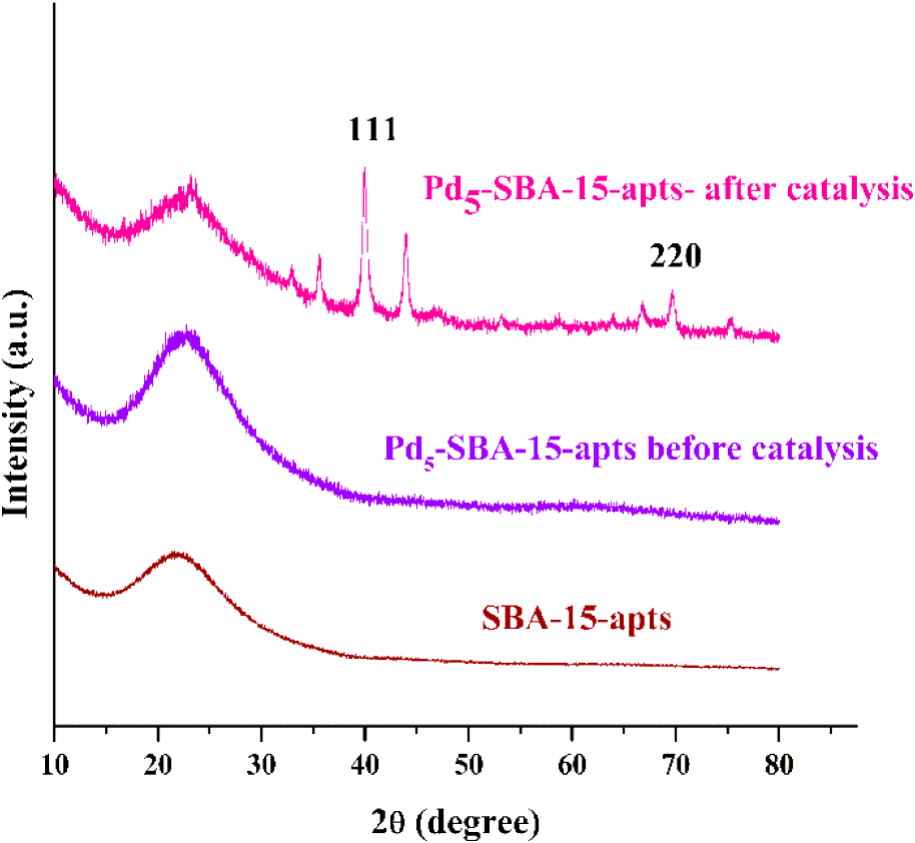
PXRD patterns of SBA-15-apts (wine), Pd_5_-SBA-15-apts before catalysis (violet), and Pd_5_-SBA-15-apts after catalysis (pink). The reflections at 2θ ≈ 40° and 68° indexed to Pd (111) and Pd (220), respectively, indicate the formation of metallic Pd nanoparticles after catalysis.

**Figure 7:**
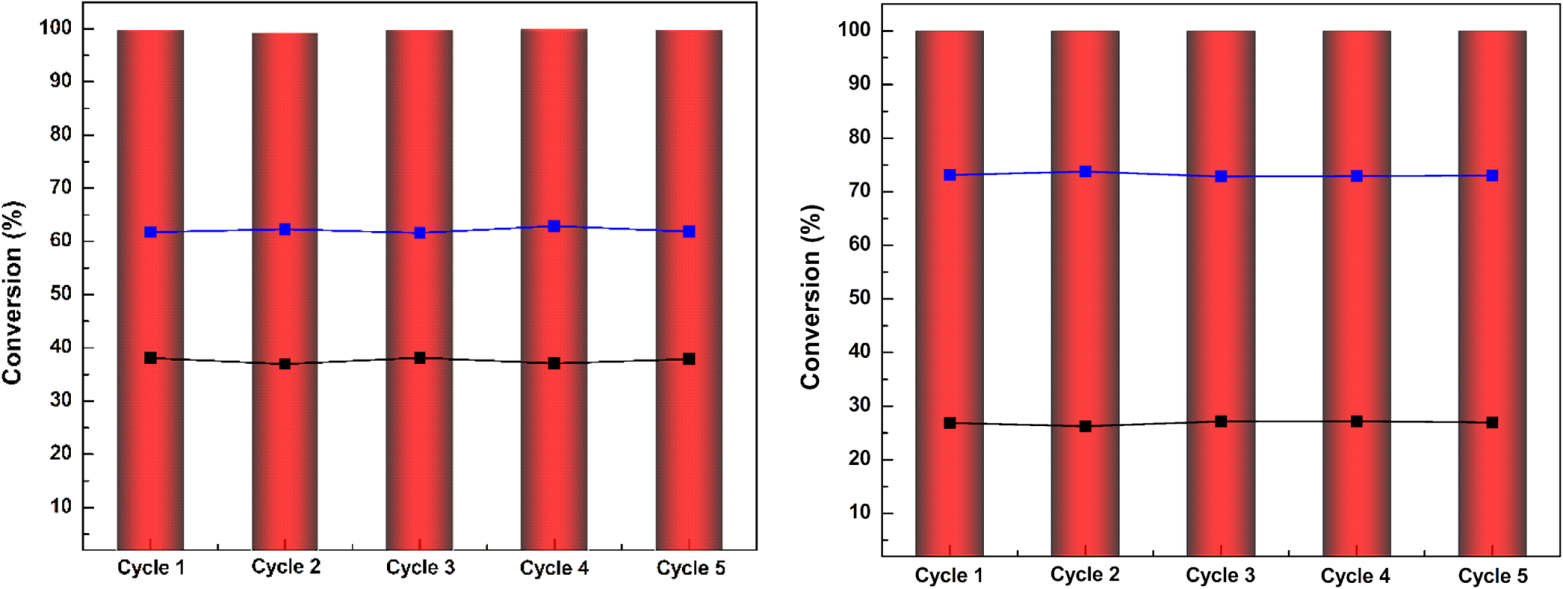
Catalyst stability over five consecutive hydroconversion cycles of *o*-xylene at 150 °C (left) and 310 °C (right) using **Na-Pd_5_** supported on modified SBA-15 and then calcined (see text for details). Reaction conditions: 28 bar H_2_, flow rate = 0.05 mL/min. Product distributions for *cis*-1,2-DMCH (black) and *trans*-1,2-DMCH (blue).

No leaching or deactivation phenomena were detected, and the catalytic performance remained reproducible throughout the recycling tests. A comparison with representative Pd-containing hydrogenation catalysts reported in the literature is provided in Table 2, highlighting the strong performance of the present Pd_5_ derived material under continuous flow conditions. In summary, **Na-Pd**_**5**_ serves as an efficient molecular precursor for generating a highly active Pd/POM-derived catalyst on SBA-15-apts for continuous flow hydrogenation of *o*-xylene. Control experiments demonstrate that simple physical mixtures of precursors used for the synthesis of **Na-Pd**_**5**_ do not reproduce catalytic performance, while post catalysis PXRD indicates formation of crystalline Pd-containing domains under reaction conditions.

**Table 2:** Comparison of Supported Polyoxopalladates or Palladium-Containing Polyoxotungstates Tested for *o*-Xylene Hydrogenation

**Ref**	**Supported catalyst**	**Treatment method** ^a^	**Conditions** ^b^	**Conv. (%)**	**S**_**c/t**_
This work	**Pd**_**5**_	M7	C6	∼100	30/70
 ^29^	**PdW**_**6**_	M7	C6	70	25/75
 ^26^	**Pd**_**13**_ **(PhAs)**_**8**_	M1	C1	36	40/60
		M2	C2	97	38/62
	**Pd**_**13**_ **As**_**8**_	M3 + M2	C1	∼100	40/60
	**Pd**_**13**_ **Se**_**8**_	M1	C1	∼100	43/57
	**MnPd**_**12**_ **(PhAs)**_**8**_	M3 + M1	C1	63	40/60
	**MnPd**_**12**_ **Se**_**8**_	M1	C1	98	42/58
	**MnPd**_**12**_ **P**_**8**_	M4	C1	∼100	43/57
		M1	C1	∼100	43/57
	**FePd**_**12**_ **Se**_**8**_	M1	C1	98	42/58
	**CoPd**_**12**_ **Se**_**8**_	M1	C1	99	40/60
	**NiPd**_**12**_ **Se**_**8**_	M1	C1	∼100	40/60
	**CuPd**_**12**_ **Se**_**8**_	M1	C1	98	43/57
	**ZnPd**_**12**_ **Se**_**8**_	M1	C1	98	42/58
	**Pd**_**15.4**_ **P**_**10**_	M4	C1	∼100	40/60
		M5	C1	∼100	40/60
		M1	C1	98	40/60
		M2	C1	∼100	40/60
	**Pd**_**15**_ **Se**_**10**_	M4	C1	87	43/57
		M5	C1	∼100	43/57
		M1	C1	97	43/57
		M2	C1	99	43/57
 ^27^	**CuPd**_**12**_ **As**_**8**_	M2	C4	∼100	43/57
		M2	C5	∼100	25/75
	**CoPd**_**12**_ **As**_**8**_	M2	C4	3	n.r.
		M2	C5	59	n.r.
^30^	**Pd**_**40**_ **-SiW**_**12**_	M6	C3	∼99	37/63

a M1 = air calcination at 550 °C for 4.5 h; M2 = air calcination at 650 °C for 4.5 h; M3 = hydrazine chemical reduction; M4 = air calcination at 300 °C for 3 h; M5 = air calcination at 400 °C for 3 h; M6 = air calcination at 250 °C for 4 h with a 0.5 °C min^–1^ ramp.; M7 = air calcination at 450 °C for 5 h with a 0.5 °C min^–1^ ramp.

b C1 = batch Parr Compact reactor, 300 °C, 90 bar H_2_, 1500 rpm; C2 = batch Parr Compact reactor, 300 °C, 120 bar H_2_, 1500 rpm; C3 = batch Parr Compact reactor, 300 °C, ∼90 bar H_2_, 1000 rpm; C4 = fixed-bed reactor, 230 °C, 28 bar H_2_, 0.05 mL min^–1^ *o*-xylene/hexane feed; C5 = fixed-bed reactor, 350 °C, 28 bar H_2_, 0.05 mL min^–1^ *o*-xylene/hexane feed. C6 = fixed-bed reactor, 310 °C, 28 bar H_2_, 0.05 mL min^–1^ *o*-xylene/hexane feed.

## Conclusions

We have synthesized the pentapalladium­(II)-containing 18-tungsto-4-phosphate [Pd_5_O_2_(HPO_4_)_2_(PW_9_O_34_)_2_]^16–^ (**Pd**_**5**_) by a conventional open-beaker synthetic procedure in aqueous solution. The polyanion **Pd**_**5**_ has a sandwich-type structure with two trilacunary **PW_9_** Keggin units encapsulating a central [Pd_5_O_2_(HPO_4_)_2_]^2+^. The title polyanion was also characterized in solution by ^31^P NMR and in the gas phase by ESI-mass spectrometry. The SAXS investigation confirms that **Pd**_**5**_ maintains its structural integrity in aqueous solution. We have also shown that **Pd**_**5**_ supported on cationically modified mesoporous SBA-15 as precatalyst, which is then calcined, resulting in the actual catalyst, is a highly active and stable and hence recyclable catalyst for the hydrogenation of *o*-xylene. In future work, we aim to synthesize derivatives of the title polyanion and study their physicochemical properties.

## Supplementary Material



## Notes

Details on catalytic reactor; sample preparation; pretreatment; catalytic studies; FT-IR; ^31^P NMR; UV−vis spectra; TGA; bond valence sum values (PDF)

## Notes

Dedicated to the memory of Dr. Bineta Keita.
