# Supplementary material for: The Phosphate-Bridged Pentapalladium(II)-Containing 18-Tungsto-4-Phosphate [Pd5O2(HPO4)2(PW9O34)2]16–: Synthesis and Physicochemical Properties
Source: Inorg Chem. 2026 Jun 25;65(27):15514–24. doi: 10.1021/acs.inorgchem.6c01326 (PMC13370861; doi:10.1021/acs.inorgchem.6c01326)
Supplement: Supplementary file 1 [file ic6c01326_si_001.pdf]

ASSOCIATED CONTENT

Supporting Information

The Phosphate-Bridged Pentapalladium(II)-Containing 18-Tungsto-4-Phosphate $[Pd_5O_2(HPO_4)_2(PW_9O_{34})_2]^{16-}$: Synthesis and Physicochemical Properties

Lakita Khidtta,[a] Ananthu Rajan,[a] Mahmoud Elcheikh Mahmoud,[a] Vinaya Siby,[a] Bassem S. Bassil,[a] Dorothea Schmidt,[a] Nikolai Kuhnert,[a] Pierre Bauduin,[b] and Ulrich Kortz[a]*

[a] School of Science, Constructor University, Campus Ring 1, 28759 Bremen, Germany.

[b] ICSM, Université de Montpellier, CEA, CNRS, ENSCM, Marcoule, 30207 Bagnols sur Cèze Cedex, France.

Email ukortz@constructor.university

Synthesis of SBA-15

SBA-15 was synthesized following a modified version of a previously reported method.[1] Specifically, 120 g of Pluronic® P123 (Mn ≈ 5800, Sigma-Aldrich) was dissolved in a solution containing 3.6 L of water and 100 mL of 37% hydrochloric acid under continuous stirring for approximately 4 hours. After complete dissolution, 270 mL of tetraethyl orthosilicate (TEOS) was added dropwise to the mixture. The resulting solution was stirred in a water bath maintained at 36 °C for 16 hours, then subjected to static aging at 95 °C for 3 days. The white precipitate formed was isolated by filtration, air-dried for two days, and finally calcined in air at 450 °C for 6 hours using a heating rate of 1 °C/min to remove the organic template.

Synthesis of modified SBA-15 (SBA-15-apts)

To prepare amine-functionalized SBA-15, 33.0 g of SBA-15 was reacted with 18 mL of (3-aminopropyl)triethoxysilane (apts) in 1.0 L of toluene under reflux for 5 hours. After cooling to room temperature, the resulting white solid was collected by filtration and subsequently dried at 100 °C for 5 hours.

Fixed-Bed Reactor for Hydrogenation Catalysis

Hydrogenation reactions were performed using a Microactivity Effi fixed-bed reactor system equipped with a 9 mm outer diameter SS316 stainless steel tubular reactor (internal diameter: 9.1 mm; length: 304.8 mm). Feed solutions were introduced using a Gilson HPLC pump. Reaction products were analyzed using an Agilent 6890 GC-FID fitted with an Rtx-DHA-100 capillary column (Restek; equivalent to DB-Petro, 100 m × 250 μm × 0.5 μm, Part No. 122-10A6). Sample injections into the gas chromatograph were performed via a CI4W.5 6-port switching valve (0.5 μL loop) from VICI.

Preparation and activation Pd_5-SBA-15-apts

To prepare the supported catalyst containing 1 wt% Pd, a total of 890.1 mg of SBA-15-apts was dispersed in 20 mL of deionized water containing 109.9 mg of **Na-Pd_5**, corresponding to a palladium content of 9.1% in the POM salt. Immobilization was performed by simple mixing of **Na-Pd_5** with SBA-15-apts in water for 30 min without intentional pH adjustment. Additional pH-dependent ^{31}P NMR experiments indicate that Na-Pd_5 remains structurally preserved under neutral to mildly basic conditions, whereas stronger acidity decreases stability. The resulting solid was isolated by filtration, air-dried, and calcined in air at 450 °C for 5 hours. The calcined catalyst was then pelletized, crushed, and sieved to obtain particles in the 40–60 mesh size range for use in fixed-bed reactor studies.

IR Spectroscopy. The FT-IR spectrum of **Na-Pd_5** was compared with that of the trilacunary POM precursor $Na_9[A\text{-}\alpha\text{-}PW_9O_{34}]\cdot 7H_2O$ (Figure S2). Intense bands at 1074 and 1012 cm^{-1} can be assigned to P-O stretching vibrations corresponding to the two distinct types of phosphate groups in the structure. The strong band at 938 cm^{-1} can be assigned to stretching vibrations of the terminal W=O bond. Bands related to stretching and bending vibrations of the W–O(W) bonds appear at 920 and 515 cm^{-1}, respectively,[2] whereas the W-O(P) stretching vibration band

appears at 839 cm^{-1}. The key differences between the IR spectra of **Na-Pd$_5$** and Na$_9$[*A*-*α*-PW$_9$O$_{34}$]·7H$_2$O are evident due to the new band around 698 cm^{-1}, likely corresponding to the stretching vibrations of the W–O–Pd bonds in **Pd$_5$**.

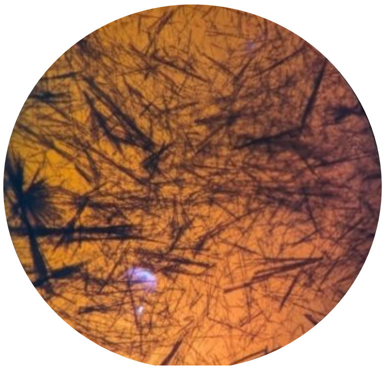

Figure S1. Single crystals (needle shaped) of **Na-Pd$_5$** under the microscope.

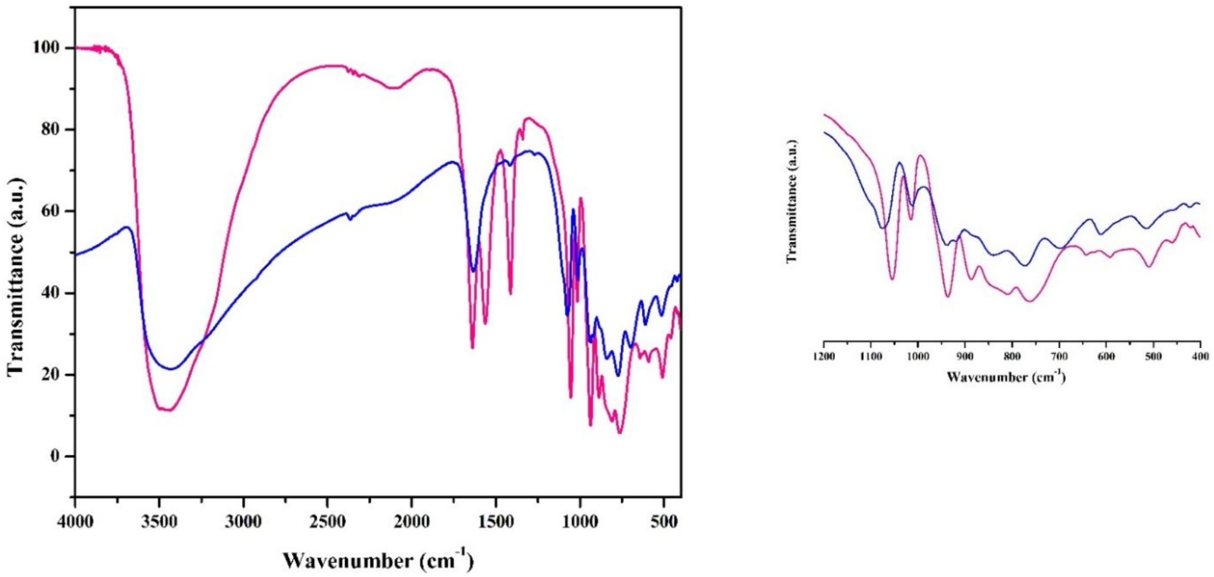

Figure S2. FT-IR spectra of **Na-Pd$_5$** (blue) and the Na$_9$[*A*-*α*-PW$_9$O$_{34}$]·7H$_2$O precursor (pink) measured on a KBr pellet.

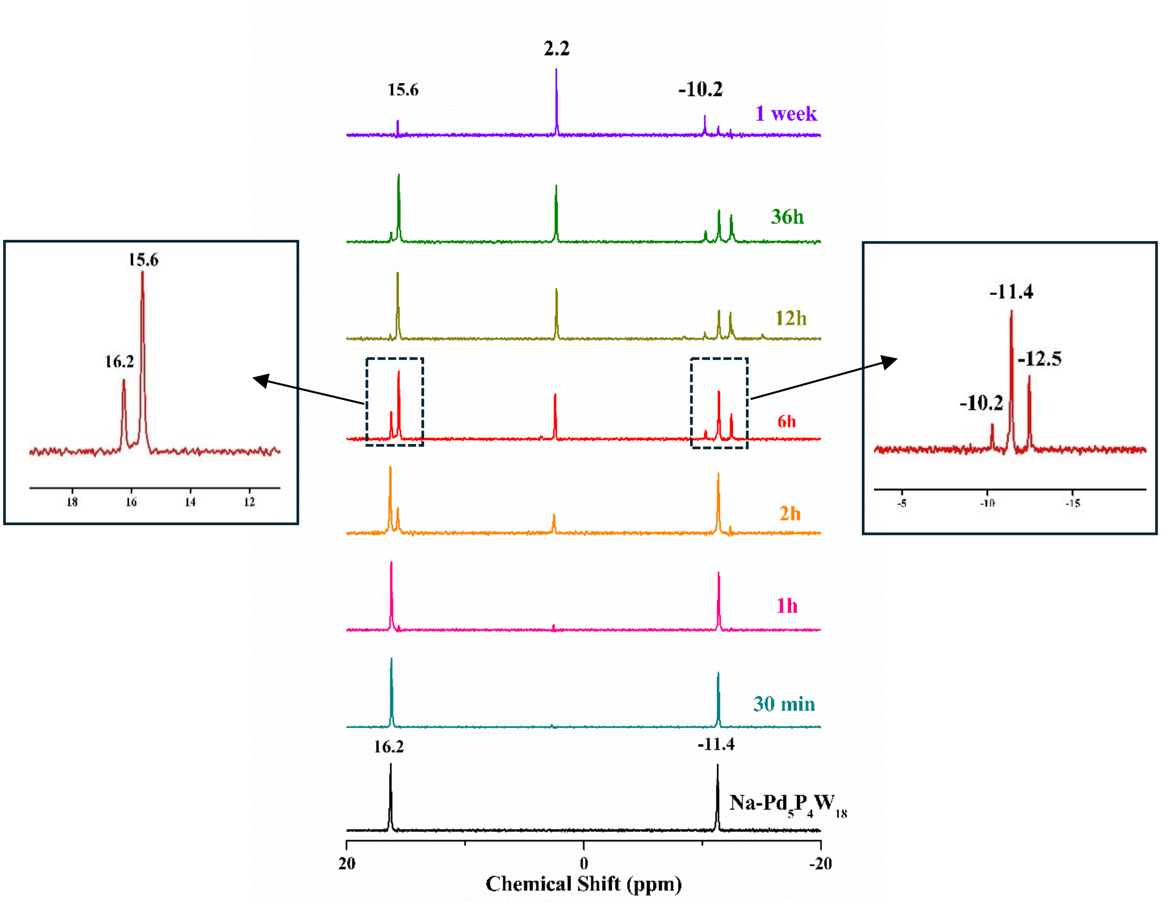

Figure S3. Time-dependent ^{31}P NMR spectra of **Na-Pd$_5$** dissolved in H_2O (pH 8.3) at room temperature.

Time-dependent ^{31}P NMR spectroscopy on **Na-Pd$_5$** (0 h → 1 week) shows that the polyanion is only partially stable in solution. At t = 0 h the spectrum shows the expected two sharp singlets at −11.4 ppm and 16.2 ppm, respectively (Figure S3). After 1 hour a weak singlet close to 0 ppm emerges, which we attribute to free phosphate, indicating the onset of polyanion decomposition. After 6 h both original peaks have broadened and resolved into multiple peaks, and the peak at 16.2 ppm has shifted to 15.6 ppm and the free phosphate signal has grown substantially. After one week the phosphate signal dominates the spectrum. Overall, time-dependent studies show that the intact **Pd$_5$** persists for roughly two hours before it progressively decomposes with the release of free phosphate in solution.

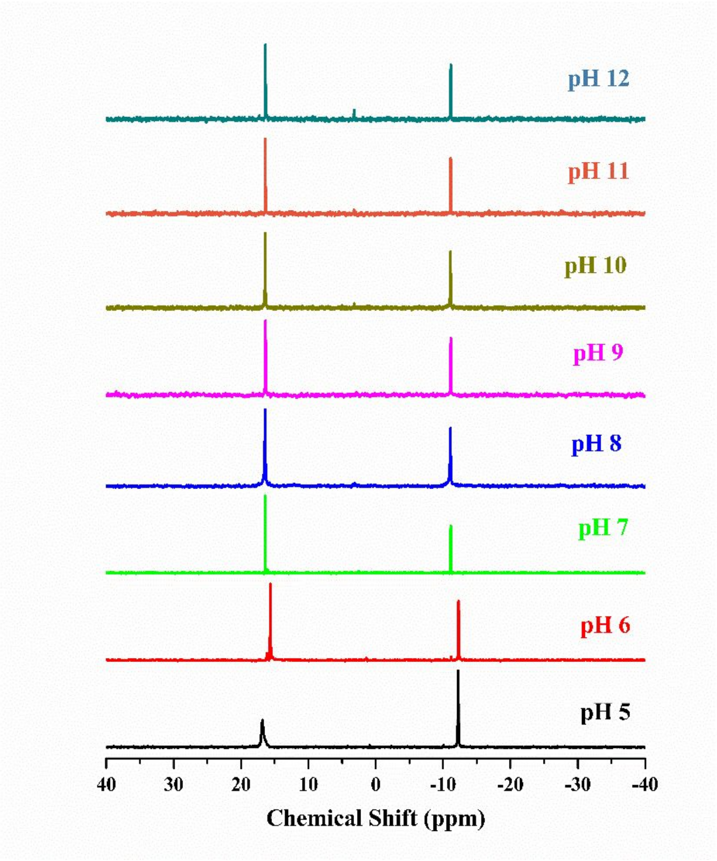

Figure S4. Time-dependent ^{31}P NMR spectra of **Na-Pd$_5$** in 0.5M sodium cacodylate solution at different pH.

The ^{31}P NMR spectra of **Na-Pd$_5$** in 0.5 M sodium cacodylate solution show the expected two characteristic resonances from pH 6 to 12, demonstrating that the polyanion **Pd$_5$** is well preserved and exhibits excellent solution stability over a broad pH range (Figure S4). At pH 5, changes in peak intensity and shape are observed, suggesting that the compound is less stable under more acidic conditions. Overall, **Na-Pd$_5$** shows good solution stability across a wide pH range.

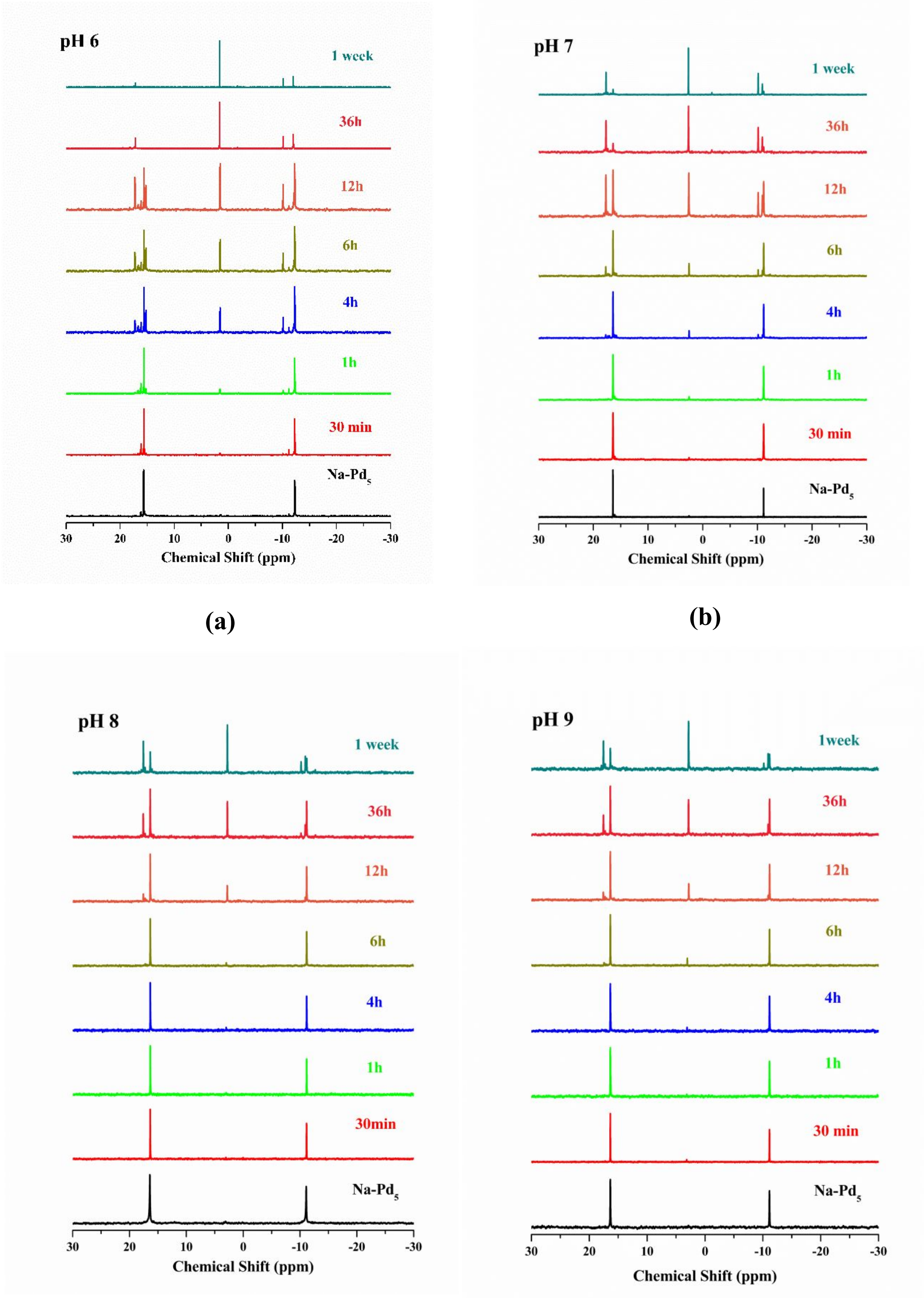

(c) (d)

Figure S5. Time-dependent ^{31}P NMR spectra of **Na-Pd$_5$** in 0.5 M sodium cacodylate solution at (a) pH 6 (top left) (b) pH 7 (top right) (c) pH 8 (bottom left) (d) pH 9 (bottom right).

Time-dependent ^{31}P NMR studies were further performed to evaluate the solution stability of **Na-Pd$_5$** at pH 6–9. At pH 6, the two characteristic resonances of **Na-Pd$_5$** are observed initially, but additional weak signals begin to appear after ca. 4 h, indicating that the compound is less stable under mildly acidic conditions. At pH 7, the two main resonances remain clearly visible for several hours, showing improved stability under neutral conditions, although minor additional signals appear after prolonged standing. At pH 8, **Na-Pd$_5$** shows better stability, with the two expected resonances remaining dominant up to at least 6 h. At pH 9, the spectra are largely preserved over the same time period, indicating that **Na-Pd$_5$** is most stable under mildly basic conditions. Overall, the time-dependent ^{31}P NMR spectra demonstrates that **Na-Pd$_5$** retains its characteristic structure for several hours, with stability increasing from pH 6 to pH 9.

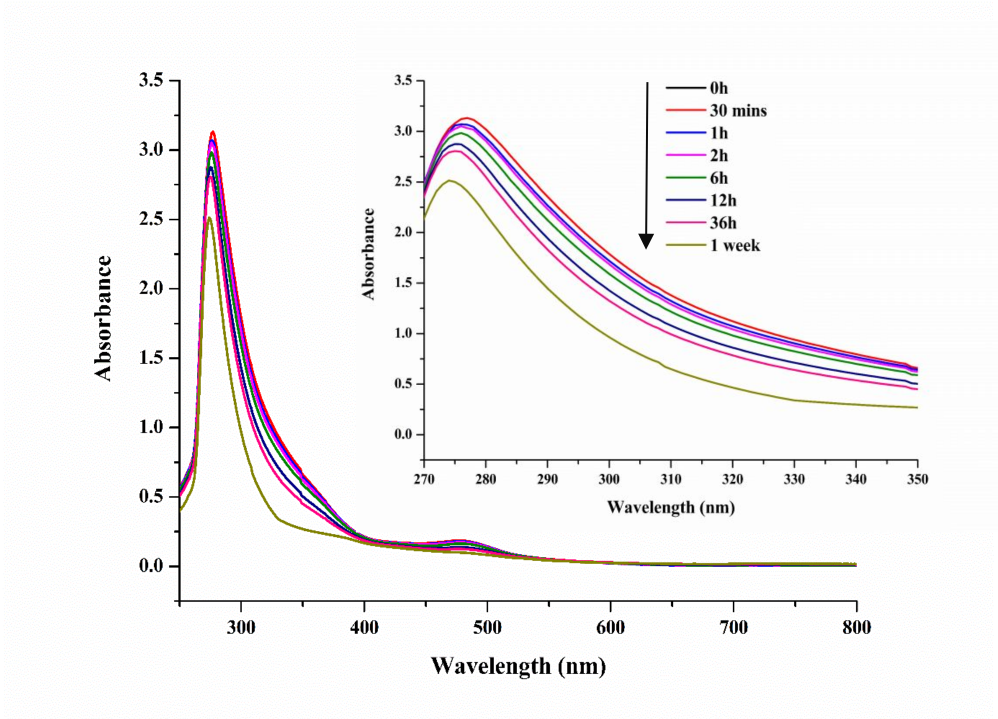

Figure S6. UV-vis spectra of **Na-Pd$_5$** in H_2O with 0.05 mM concentration. The time-dependent UV-vis spectra showed shows a shift of the absorption maximum from 278 to 275 nm.

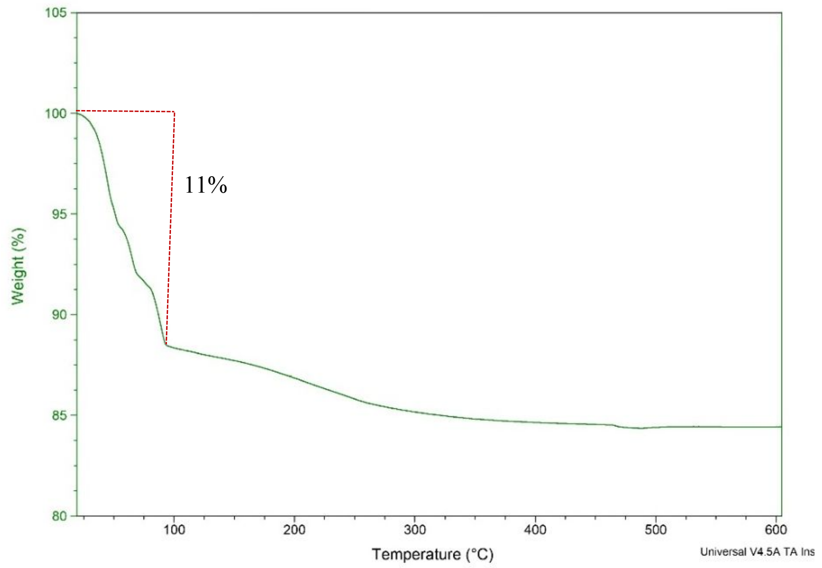

Figure S7. Thermogram of **Na-Pd$_5$** from room temperature to 600 °C under N_2 showing ca. 11% weight loss from room temperature to ca 100 °C, corresponding to ~37 H_2O molecules.

The TGA curve of **Na-Pd$_5$** shows an initial weight loss up to ca. 100 °C, corresponding to the loss of lattice water molecules (~11%; Figure S7). Based on this value, the water content is estimated to be approximately 37 H_2O molecules per formula unit, which differs from the value obtained from elemental analysis (25 H_2O), likely due to differences in the degree of sample drying prior to analysis. The second weight-loss step between ca. 100 and 600 °C is attributed to decomposition of the polyanion.

Table S1. Bond valence sum (BVS) values for the two types of μ_3 bridging oxo ligands in the central palladium-oxo unit of **Pd$_5$**. Bond valence (v_{ij}) = exp[(R_{ij}-d_{ij})/b (R_{ij} = bond valence parameter; b = 0.37); bond valence sum (V_i) = $\Sigma_j v_{ij}$.

**μ_3-O**	**Bond distance (Å)**	**BVS value**
O135	Pd1-O135(1.9742)	1.801
	Pd3-O135 (1.9797)	
	Pd5-O135 (1.9888)	
O245	Pd2-O135(1.9604)	1.802
	Pd4-O135(1.9758)	
	Pd5-O135(2.0071)	

Table S2. Bond valence sum (BVS) values for different types of μ_2 bridging oxo ligands in the central palladium-oxo unit of **Pd$_5$**.

**μ_2-O**	**Bond distance (Å)**	**BVS value**	**μ_2-O**	**Bond distance (Å)**	**BVS value**
O13P	P3-O13P (1.5566)	1.745	O33P	P3-O33P (1.5338)	1.791
	Pd1-O13P (2.0014)			Pd3-O33P (2.0204)	
O35P	P3-O35P (1.5417)	1.750	O44P	P4-O44P (1.5544)	1.718
	Pd5-O35P (2.0307)			Pd4-O44P (2.0239)	
O45P	P4-O45P (1.5496)	1.727	O24P	P4-O24P (1.5649)	1.697
	Pd5-O45P (2.0289)			Pd2-O24P (2.0157)	

Table S3. Bond valence sum (BVS) values for the monoprotonated oxygen atoms (OH) in the central palladium-oxo unit of **Pd$_5$**.

**Protonated oxygen (OH)**	**Bond distance (Å)**	**BVS value**
O1P3	P3-O1P3 (1.5380)	1.238
O1P4	P4-O1P4 (1.5154)	1.315

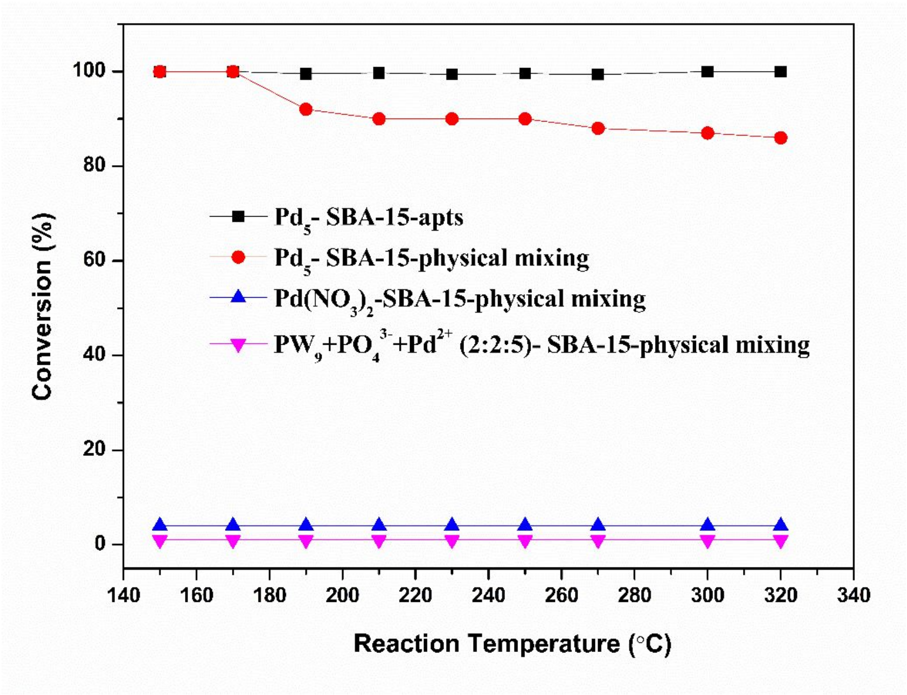

Figure S8. Control experiments for *o*-xylene hydrogenation using Pd_5-SBA-15-apts (black), Pd_5-SBA-15-physical mixing (red), $Pd(NO_3)_2$-SBA-15-physical mixing (blue) and PW_9+PO_4^{3-} +Pd^{2+} (2:2:5)- SBA-15-physical mixing (pink).

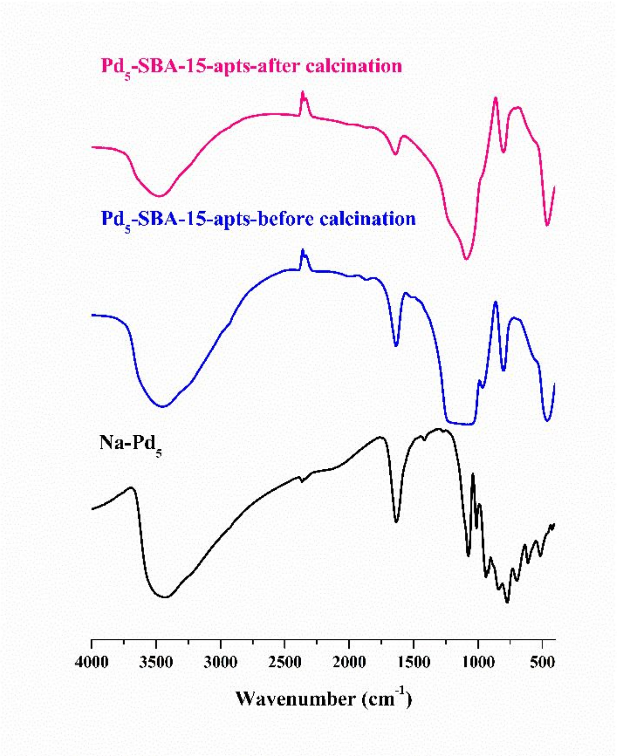

Figure S9. FT-IR spectra of **Na-Pd$_5$** (black), Pd_5-SBA-15-apts before calcination (blue), and Pd_5-SBA-15-apts after calcination (pink).

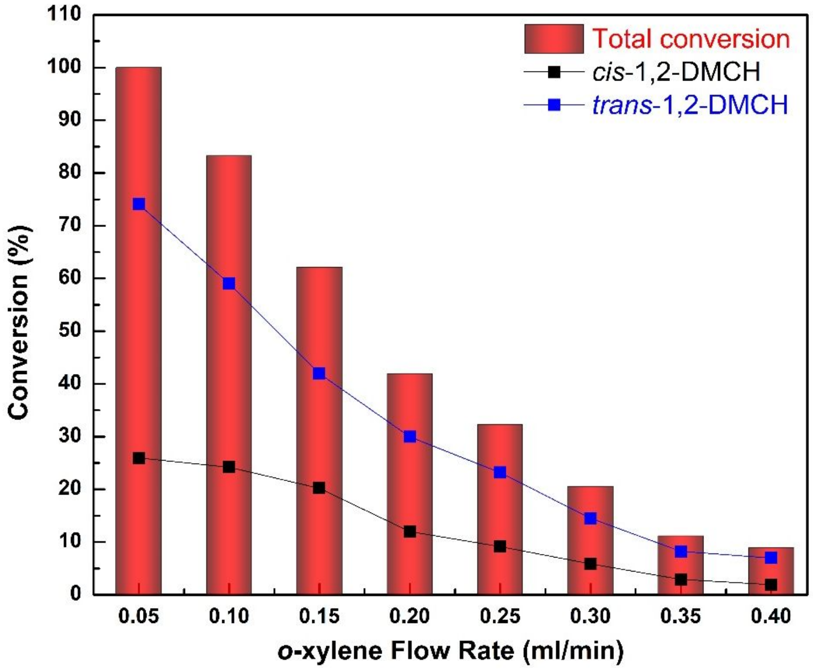

Figure S10. Effect of flow rate on the product distribution during the hydroconversion of *o*-xylene at 310 °C using the Pd_5-SBA-15-apts catalyst after pretreatment. Reaction conditions: 28 bar H_2. Product selectivity shown for *cis*-1,2-DMCH (black) and *trans*-1,2-DMCH (blue).

References

1. (a) Zhao, D.; Feng, J.; Huo, Q.; Melosh, N.; Fredrickson, G. H.; Chmelka, B. F.; Stucky, G. D. Triblock Copolymer Syntheses of Mesoporous Silica with Periodic 50 to 300 Angstrom Pores. *Science*. **1998**, *279*, 548–552. (b) Zhao, D.; Huo, Q.; Feng, J.; Chmelka, B. F.; Stucky, G. D. Nonionic Triblock and Star Diblock Copolymer and Oligomeric Surfactant Syntheses of Highly Ordered, Hydrothermally Stable, Mesoporous Silica Structures. *J. Am. Chem. Soc.* **1998**, *120*, 6024–6036.
2. (a) Rocchicchioli-Deltcheff, C.; Fournier, M.; Franck, R.; Thouvenot, R. *Inorg. Chem.* **1983**, *22*, 207–216. (b) Thouvenot, R.; Fournier, M.; Franck, R.; Rocchicchioli-Deltcheff, C. *Inorg. Chem.* **1984**, *23*, 598–605
